# Taming the transplant troll: Exploring racial and ethnic disparities in cytomegalovirus infection among kidney transplant patients

**DOI:** 10.1371/journal.pone.0317383

**Published:** 2025-01-29

**Authors:** M. Gabriela Cabanilla, Ashlee Dauenhauer, Briana St John, Deirdre Hill, Joshua Larson

**Affiliations:** 1 Division of Infectious Diseases, Department of Internal Medicine, University of New Mexico Health Sciences Center, Albuquerque, NM, United States of America; 2 Department of Pharmacy, University of New Mexico Health Sciences Center, Albuquerque, NM, United States of America; 3 Division of Transplant Nephrology, Department of Internal Medicine, University of New Mexico Health Sciences Center, Albuquerque, NM, United States of America; 4 University of New Mexico School of Medicine, Albuquerque, NM, United States of America; University of St Andrews, UNITED KINGDOM OF GREAT BRITAIN AND NORTHERN IRELAND

## Abstract

**Background:**

Cytomegalovirus (CMV) infection poses a significant risk to kidney transplant recipients. This study investigated CMV disease incidence, outcomes, and management challenges in racial and ethnic minority populations following kidney transplantation.

**Methods:**

This single-center, mixed-methods study included a retrospective cohort analysis of kidney transplant recipients (n = 58) and qualitative surveys of healthcare providers. Patients were categorized as minorities (n = 49) or non-Hispanic whites (n = 9). The primary outcome was CMV disease incidence. Secondary outcomes included graft failure, mortality, and identification of management barriers.

**Results:**

The cumulative incidence of CMV disease was higher in minorities than in non-Hispanic whites (12.3% vs. 0%, p = 0.58), although the difference was not statistically significant. All graft failures (8.6%, n = 5) occurred in the minority group. Although not statistically significant, all-cause mortality was higher in the minority group (24.5% vs. 11.1%, p = 0.54), with 46.2% of the deaths occurring within 90 days of CMV diagnosis. Qualitative analysis revealed challenges in diagnosis, treatment-related side effects, medication costs, and insurance barriers. The providers emphasized the importance of interdisciplinary collaboration and standardized protocols.

**Conclusion:**

While limited by the small sample size, this study highlights potential disparities in the incidence and outcomes of CMV disease among minority kidney transplant recipients, suggesting that barriers in care and access may contribute to these differences. These hypothesis-generating findings underscore the need for larger multicenter studies to validate these patterns and to inform targeted strategies that may reduce inequities in post-transplant outcomes.

## Introduction

The effects of race and ethnicity on kidney transplantation have been topics of interest for many years. Several studies have shown that racial and ethnic minority candidates are less likely to advance through the stages of kidney transplant evaluation necessary to receive a transplant [1–3]. These findings are an example of the well-established, more generalized disparity in access to care among racial and ethnic minority populations, which has raised concerns regarding potential barriers to the treatment of opportunistic infections after transplantation. One such infection is cytomegalovirus (CMV), which is associated with high morbidity and mortality after kidney transplantation, including graft failure [4, 5].

The overall CMV seroprevalence in the United States is 50%, although this varies depending on age, geography, and socioeconomic status [6, 7]. The incidence of CMV infection after kidney transplantation has been reported as 8–32% [8], with higher infection rates observed among certain minority populations [9, 10]. Although previous studies have shown significant morbidity and mortality associated with CMV infection following kidney transplantation, there remains a critical gap in understanding the specific consequences and outcomes of this infection among racial and ethnic minority populations. Additionally, the extent to which these disparities translate into differential clinical outcomes and the identification of modifiable risk factors in this context remains unclear. This knowledge gap presents a critical challenge in the pursuit of equitable post-transplant care for all recipients, irrespective of their racial or ethnic backgrounds. Balfour originally described CMV as a “transplant troll,” symbolizing its insidious and unpredictable nature in the transplant setting [11]. In this study, we extended this metaphor to highlight how systemic inequities compound the burden of CMV infection among racial and ethnic minority transplant recipients. Therefore, we aimed to investigate the unique challenges and disparities faced by these populations in the context of CMV infection after kidney transplantation.

## Methods

### Study design

This single-center, mixed-methods study employed both quantitative and qualitative approaches to obtain a comprehensive understanding of CMV infection in racial and ethnic minority populations following kidney transplantation. Quantitative data were collected through a retrospective comparative cohort study of kidney transplant recipients at the University of New Mexico Health Sciences Center (UNM HSC) between January 1, 2012, and July 31, 2022. Patients were classified into two groups based on their racial and ethnic backgrounds: minorities (including Hispanics, Native Americans, and others) and non-Hispanic whites, who served as the reference population. The data are presented in accordance with the STROBE guidelines for reporting observational studies [12]. Data for the retrospective portion of this study was accessed from January 1, 2023, through October 1^st^, 2023. During the data collection process, the authors MGC, AD, and BSJ had access to information that could identify individual patients. After data collection, all identifying information was secured to a password-protected computer in a locked office to maintain patient confidentiality.

The qualitative component of the study involved anonymous, open-ended survey questions administered through the Research Electronic Data Capture platform (REDCap) hosted at UNM HSC [13, 14] from October 1st, 2023, through November 1st, 2023. REDCap is a secure web-based software platform designed to support data capture for research studies [13, 14]. The survey was designed to collect qualitative data through open-ended questions and prompts to explore the participants’ experiences, perceptions, and challenges related to CMV management after kidney transplantation. Open-ended questions allowed for the collection of narrative data and personal insights. The survey was reviewed by two infectious diseases experts prior to distribution to ensure its content validity and relevance to the study objectives.

This study was approved by the Human Research Review Committee of the UNM HSC (study #22–321). The requirement for informed consent for the quantitative part of this study was waived owing to the retrospective nature of the study. For the qualitative component, written informed consent was obtained from all the participants.

### Study population, recruitment and data collection

UNM HSC is a publicly funded tertiary care center in Albuquerque, New Mexico, serving as the primary safety net hospital for the state’s underserved population [15]. Patients were identified through the Organ Transplant Tracking Record (OTTR) database using specific criteria. Clinical data on demographics, comorbidities, transplant details, CMV prophylaxis, diagnosis, treatment, and outcomes were extracted from medical records. To enhance accuracy, data from laboratory reports, progress notes, and pharmacy records were cross-referenced, and a standardized collection form was used to minimize errors during entry.

Patients were included if they were ≥ 18 years old, had undergone kidney transplantation within the specified time frame, and had documented CMV infection via PCR post-transplantation. Exclusion criteria included missing race/ethnicity data or incarceration.

Survey participants were recruited from UNM HSC providers with expertise in transplantation, nephrology, internal medicine, or infectious diseases, and were ≥ 18 years old. Survey responses were anonymized to protect privacy.

### Study outcomes and definitions

The primary outcome of this study was to evaluate the incidence of CMV disease among racial and ethnic minority populations at any time following kidney transplantation compared to non-Hispanic white recipients. Secondary outcomes included evaluation of clinical endpoints of CMV infection (i.e., graft failure and mortality) and identification of barriers and facilitators in CMV management among healthcare providers.

CMV disease was defined based on the updated criteria proposed by Ljungman et al [16]. These criteria distinguish between CMV infection, CMV syndrome, and CMV end-organ disease, with emphasis on the specific clinical and diagnostic features of each category in transplant patients. Study investigators determined whether a diagnosis of proven CMV disease was made during the patient encounter using these definitions. CMV infection was defined as the presence of CMV replication in any body fluid or tissue specimen, regardless of symptomatology, as detected using PCR. CMV viremia was defined as a detectable CMV viral load via whole-blood PCR, with a detectable range of >34.5 IU/mL.

### Statistical analysis

Descriptive statistics were used to characterize the subjects by demographics, transplant details, and prophylaxis, according to race and ethnicity. Transplant and other information were compared by combining all minorities compared to non-Hispanic whites using a chi-square test with calculation of Fisher’s exact p-value for expected cell counts of less than five. Similar analyses were conducted for the risk factors of CMV disease, cumulative CMV disease incidence and all-cause mortality using the chi-square and Fisher’s exact tests. All statistical tests used a two-sided alpha = .05. All analyses were conducted using Statistical Analysis Systems (SAS) software, v. 9.4 (Cary, N. Carolina).

Survey responses were collected and stored securely on the REDCap platform. Qualitative data were exported for thematic analysis. The responses were de-identified and analyzed. Thematic analysis involved the coding, categorization, and identification of common themes and patterns within the narrative responses.

## Results

### Quantitative findings

A total of 58 kidney transplant recipients were included in the study, of which 49 (84.5%) were classified into the minority group, and nine (15.5%) into the non-Hispanic white group. The mean age of the minority group was 57.9 years (SD 15.9), while the non-Hispanic white group had a mean age of 62 years (SD 14). Among the minority group, 26 (53.1%) were of Hispanic ethnicity, 20 (40.8%) were Native American, and three (6.1%) belonged to other ethnic minority groups. The baseline characteristics of the cohort (Table 1) were well-balanced, except for an overall higher Charlson comorbidity index in the minority group. The most common comorbidity in the cohort was diabetes (43.1%). The most common organ used in transplantation was from a deceased donor (91.4%, n = 53), and most patients received transplants from 2020 to 2022 (58.6%, n = 34), without any significant differences between the groups.

**Table 1:** Baseline characteristics of kidney transplant recipients with CMV infection.

Characteristics	Minorities^†^ (n = 49)	Non-Hispanic whites (n = 9)	P-value ^‡^
**Age in years**, mean (SD)	57.9 (15.9)	62 (14.0)	0.48
**Male sex**, n (%)	25 (51.0)	4 (44.4)	1.0
**Charlson comorbidity index**, n (%)			0.01
0	7 (14.3)	1 (11.1)	
1	4 (8.2)	1 (11.1)	
2	5 (10.2)	5 (55.6)	
≥ 3	33 (67.3)	2 (22.2)	
**Common comorbidities, n (%)**			0.0504
Diabetes	24 (48.9)	1 (11.1)	
Peripheral vascular disease	8 (16.3)	0	
Heart failure	6 (12.2)	0	
Other ^§^	6 (12.2)	0	
**Year of transplantation**, n (%)			0.50
2010–2014	2 (4.1)	0	
2015–2019	17 (34.7)	5 (55.5)	
2020–2022	30 (61.2)	4 (44.4)	
**Donor organ type**, n (%)			0.17
Deceased	46 (93.9)	7 (77.8)	
Living	3 (6.1)	2 (22.2)	
**CMV serologies**, n (%)			0.23
D+/R+	25 (51.0)	2 (22.2)	
D+/R-	10 (20.4)	4 (44.4)	
D-/R+	6 (12.2)	2 (22.2)	
D-/R-	0	0	
Unknown	8 (16.3)	1 (11.1)	
**CMV viral load in IU/mL**, n (%)			0.49
< 200	21 (42.9)	6 (66.7)	
200–9,999	21 (42.9)	2 (22.2)	
≥ 10,000	7 (14.3)	1 (11.1)	
**CMV prophylaxis and duration**^¶^, n (%)			0.09
Valganciclovir			
3 months	30 (61.2)	4 (44.4)	
6 months	11 (22.5)	3 (33.3)	
Valacyclovir			
3 months	0	0	
6 months	0	1 (11.1)	
Acyclovir			
3 months	0	0	
6 months	1 (2.0)	0	
None	2 (4.1)	0	
Unknown	5 (10.2)	1 (11.1)	

^†^Minorities represented in the sample comprised 26 Hispanics, 20 Native Americans, two African Americans, and one Asian participant.

^‡^Fisher’s exact test was utilized for expected cell counts < 5.

^§^Other comorbidities included myocardial infarction (n = 1), dementia (n = 1), connective tissue disease (n = 1), liver disease (n = 2), and non-metastatic tumors (n = 1).

^¶^Refers to initial CMV prophylaxis only and does not account for changes while on therapy.

Abbreviations: D-, donor negative; D+, donor positive; R-, recipient negative; R+, recipient positive; SD, standard deviation.

The cumulative incidence of CMV disease was higher in the minority group than that in the non-Hispanic white group (12.3% vs. 0%, p = 0.58), although not statistically significant. Three patients had biopsy-proven CMV colitis and three had PCR-proven CMV pneumonitis. The median viral load for patients with CMV disease was 3,107 IU/mL (IQR 203, 8215). Two patients were initially treated with ganciclovir, while three received valganciclovir. For unclear reasons, one patient received no initial treatment and weekly serum PCR monitoring was performed. Additional management strategies employed for the treatment of CMV disease included modifications of immunosuppression. Antimetabolites were suspended in three patients. Antimetabolites and calcineurin inhibitors were suspended in one patient. Calcineurin inhibitors alone were suspended in one patient, and the antimetabolite dose was decreased in one patient.

CMV prophylaxis was administered to 86.2% (50/58) of the patients in the cohort, with the most common initial prophylactic agent being valganciclovir (96%). One patient in the minority group received acyclovir and one patient in the non-Hispanic white group received valacyclovir. Prophylaxis dosing was inappropriate for 20% (10/50) of the patients due to suboptimal dosing based on estimated creatinine clearance at the time of prophylaxis initiation. Although a higher proportion of non-Hispanic white patients received inappropriate prophylaxis dosing than minority patients (37.5% vs. 16.7%), this difference was not statistically significant (p = 0.13). Prophylaxis status was unknown in six patients: five in the minority group and one in the non-Hispanic white group.

Early discontinuation of prophylaxis was observed in 19 patients: 15 in the minority group and four in the non-Hispanic white group. The most common reason for discontinuing prophylaxis was leukopenia (78.9%, n = 15). Other reasons for discontinuation of prophylaxis included switching to treatment for viremia (n = 3) and transitioning to comfort measures (n = 1). In cases of discontinuation due to leukopenia, alternative prophylaxis was initiated in four patients (two in the minority group and two in the non-Hispanic white group), while weekly PCR monitoring was performed for the other patients. Alternative prophylaxis regimens used in the four patients included letermovir (n = 2, both in the non-Hispanic white group) and valacyclovir (n = 2, both in the minority group). Adverse events occurred in 30% (15/50) of patients receiving prophylaxis, with 12 cases in the minority group (28.6%) and three in the non-Hispanic white group (37.5%). Despite the apparent differences in the proportions, this variation was not statistically significant (p = 0.38).

Graft failure occurred in 8.6% (n = 5) of the patients, all in the minority group (p = 0.24). Of these patients, one was diagnosed with CMV disease and received no treatment, resulting in graft loss 14 months after CMV disease diagnosis. CMV management in this patient included antimetabolite suspension and weekly PCR monitoring.

Thirteen patients died during the study period. All-cause mortality differed between the minority group (24.5%, n = 12) and non-Hispanic white group (11.1%, n = 1), although this difference was not statistically significant (p = 0.54). Of these patients, 46.2% died within 90 days of CMV diagnosis, of which 83.3% were in the minority group. Patients with diabetes had a significantly higher cumulative mortality than those without diabetes (40% vs. 9%, respectively, p = 0.01). Study outcomes have been stratified based on donor and recipient CMV serologies in Table 2. Table 3 summarizes the clinical features, diagnostic criteria, and management strategies of patients with CMV end-organ disease observed in our cohort.

**Table 2:** Study outcomes stratified by donor/recipient CMV serology status and ethnicity.

CMV serologies	Ethnicity, n	CMV disease incidence ^†^ (n)	P-value ^‡^	Graft failure ^†^ (n)	P-value ^‡^	All-cause mortality ^†^ (n)	P-value ^‡^
**D+/R+**	Minorities, 25	0.16 (4)	1.0	0.08 (2)	1.0	0.2 (5)	1.0
	Non-Hispanic Whites, 2	0 (0)		0 (0)		0 (0)	
**D+/R-**	Minorities, 10	0 (0)	-	0 (0)	-	0.3 (3)	1.0
	Non-Hispanic Whites, 4	0 (0)		0 (0)		0.25 (1)	
**D-/R+**	Minorities, 6	0 (0)	-	0.17 (1)	1.0	0.5 (3)	0.46
	Non-Hispanic Whites, 2	0 (0)		0 (0)		0 (0)	
**D-/R-**	Minorities, 0	-	NA	-	NA	-	NA
	Non-Hispanic Whites, 0	-		-		-	
**Unknown**	Minorities, 8	0.25 (2)	1.0	0.25 (2)	1.0	12.5 (1)	1.0
	Non-Hispanic Whites, 1	0 (0)		0 (0)		0 (0)	

^†^Refers to cumulative incidence.

^‡^Fisher’s exact test was utilized for expected cell counts < 5.

Abbreviations: D-, donor negative; D+, donor positive; NA, not applicable; R-, recipient negative; R+, recipient positive.

**Table 3:** Clinical features, diagnostic criteria, and management of CMV end-organ disease.

Organ or System	Number of Patients	Clinical Features	Diagnostic Criteria	Management
**Gastrointestinal**	3	Diarrhea, abdominal pain, bleeding, weight loss	Biopsy-proven CMV inclusions	• Antivirals (valganciclovir, ganciclovir) • Antimetabolites suspended in 2 cases
**Pulmonary**	3	Cough, dyspnea, hypoxia	PCR-proven CMV in bronchoalveolar lavage and new infiltrates on imaging	• Antivirals (valganciclovir, ganciclovir) • Antimetabolites suspended in 2 cases

Abbreviations: CMV, cytomegalovirus; PCR, polymerase chain reaction.

### Qualitative findings

A thematic analysis of healthcare provider surveys revealed several key challenges in managing CMV in kidney transplant recipients. These included difficulties in diagnosis, treatment-related side effects (primarily leukopenia), medication costs and insurance barriers (Table 4). Providers identified facilitators of effective management such as subspecialty consultations, new therapeutic options, and pharmacist involvement. Monitoring practices varied, with most relying on serial PCR tests. Treatment strategies commonly involved antiviral medications and reduction of immunosuppression, with growing interest in newer prophylactic agents such as letermovir. Systemic challenges included coordination of care between specialties and ensuring appropriate follow-up. Providers suggested improvements such as standardized protocols, better patient tracking systems, and enhanced interdisciplinary collaboration.

**Table 4:** Key themes identified in healthcare provider surveys on CMV management in kidney transplant recipients.

Main Theme	Sub-Themes	Representative Quote
**Challenges in CMV management**	Diagnosis difficulties	“Diagnosis is a challenge because it often requires tissue confirmation.”
	Treatment-related side effects	“Leukopenia and GI side effects limiting antiviral treatments available.”
	Medication access barriers	“Cost and side effects (leukopenia, GI).”
	Insurance-related issues	“Insurance approval of new therapies (letermovir).”
**Facilitators of effective management**	Subspecialty consultations	“Availability of ID consultation.”
	New therapeutic options	“New therapies that have become available.”
	Pharmacist involvement	“Presence of pharmacists to help with transitions of care, prescription cost and renal dosing.”
**Monitoring practices**	Serial PCR testing	“If a patient has CMV viremia we check PCR levels weekly until they clear it.”
	Varied screening approaches	“We do not routinely screen for CMV. Instead, we order a CMV PCR only when clinical suspicion arises.”
**Systemic challenges**	Care coordination	“A more integrated Nephrology-Infectious diseases care approach.”
	Follow up and monitoring	“Following up with patients to ensure no loss to follow up and that they are getting labs appropriately.”
	Insurance approval for new therapies	“Insurance companies should work together with pharma to establish reasonable market access approaches.”
**Importance of interdisciplinary collaboration**	Nephrology-Infectious diseases cooperation	“ID involvement if a patient is requiring hospital admission.”
	Pharmacist integration	“Pharmacist involvement in treatment and monitoring.”
**Suggestions for improvement**	Standardized protocols	“Establish and follow a treatment protocol.”
	Enhanced tracking systems	“A better way to track patients that we are treating for CMV, to ensure nobody falls through the cracks.”
	Improved insurance coverage	“Social work involvement if cost is an issue for patients.”

Abbreviations: CMV, cytomegalovirus; GI, gastrointestinal; ID, infectious diseases; PCR, polymerase chain reaction.

## Discussion

This mixed-methods study revealed important findings and challenges in managing CMV infections among minority kidney transplant recipients. Our quantitative analysis showed a higher cumulative incidence of CMV disease in the minorities than in non-Hispanic whites (12.3% vs. 0%, p = 0.58), although this difference was not statistically significant. Differences in graft failure and all-cause mortality between groups were also not statistically significant but all graft failures occurred in the minority group, and all-cause mortality was higher in this group, with a substantial proportion of deaths occurring within 90 days of CMV diagnosis. Qualitative insights from healthcare providers highlighted complexities in managing CMV in kidney transplant recipients.

The increased CMV disease incidence in minority recipients aligns with previous studies reporting higher rates of CMV infection and complications in minority populations following transplantation [17–21]. This potential disparity could be attributed to several factors, including differences in CMV seroprevalence, socioeconomic status, and access to health care resources [19, 22, 23]. Our local experience suggests that minority populations tend to have decreased access to anti-CMV medications, often because of cost limitations, along with formulary restrictions or delays in insurance approval. Additionally, lower medication adherence and health literacy, along with challenges in performing timely laboratory testing or attending clinic appointments owing to unreliable transportation, may contribute to delays in diagnosis and treatment. These socioeconomic and logistical challenges likely compounded the medical complexities of managing CMV disease, potentially explaining the higher incidence and poorer outcomes in minority kidney transplant recipients.

Appropriate prophylaxis selection and duration are critical for mitigating CMV risk. The decision between prophylaxis durations of 100 and 200 days should be determined through risk stratification using CMV serostatus, with a longer duration of 200 days recommended for high-risk patients (i.e., D+/R-) [24]. In our study, the most common reason for discontinuation of prophylaxis was leukopenia (78.9%). To address this, our center typically discontinues therapy and monitors weekly serum CMV PCR, a practice recommended by previous studies [25–27]. In a small number of patients, the alternative approaches included switching to valacyclovir (n = 2) or letermovir (n = 2). Interestingly, valacyclovir was used as an alternative prophylactic agent exclusively in minority patients, while letermovir was utilized in non-Hispanic whites. This disparity is likely due to insurance barriers; however, letermovir has been shown to be superior to high-dose valacyclovir for CMV prophylaxis, with a reduced CMV incidence and improved event-free survival [28]. These observations highlight the need for personalized prophylaxis strategies that consider patient-specific factors and are reevaluated based on medication tolerance and adherence barriers.

The treatment approaches for CMV management varied considerably in our cohort. In the absence of a formal protocol, treatment strategies varied depending on provider and individual patient factors. These strategies included initiating antiviral medication, reducing immunosuppression, or a combination of both. The choice of antiviral agent was influenced by factors such as disease severity, organ involvement, CMV PCR trends, and patient-specific considerations such as medication accessibility and affordability. This variability highlights the need for standardized protocols while maintaining flexibility to address individual patient needs.

The higher proportion of graft failure observed exclusively in the minority group is concerning and consistent with previous studies documenting disparities in transplant outcomes among racial and ethnic minority groups [29, 30]. Kidney allograft loss occurs through a complex interplay of multiple factors, including age, socioeconomics, time on dialysis before transplantation, environmental factors, center-specific therapies, and etiology of end-stage renal disease (ESRD) [31, 32]. Among the five patients identified with CMV disease and subsequent graft failure, the factors surrounding their kidney transplants and subsequent graft loss were diverse. Notable differences among these patients included the time since transplantation, etiology of ESRD, history of rejection, and comorbidities. Minority status was a common factor linking patients in our cohort. Although our study could not establish causality, the temporal relationship between CMV disease and graft failure in one case, as well as the higher incidence of graft failure exclusively in the minority group suggests a potential link that merits further exploration.

The increased all-cause mortality in the minority group (although not statistically significant), particularly within 90 days of CMV diagnosis, highlights the potential severity of the CMV-related complications in this population. As with graft loss, the factors affecting mortality risk after transplantation are multifactorial. Malignancy, cardiovascular disease, and infection are the most common causes of mortality among renal transplant recipients, with patient age, ESRD due to diabetes, and pre-transplant dialysis as common risk factors [33]. CMV disease has also been identified as a key mortality risk factor among Latin-American populations [34]. Although non-statistically significant, our study observed an increase in all-cause mortality in the minority group, particularly within 90 days of CMV diagnosis, and diabetes was a primary risk factor in this group. Diabetes, whether pre-existing prior to transplantation or post-transplantation, is associated with increased mortality in renal transplant recipients [35]. The significantly higher mortality proportion among patients with diabetes underscores the importance of considering comorbidities for risk stratification and management.

Qualitative analysis of healthcare provider surveys revealed several key challenges in managing CMV in kidney transplant recipients. These included difficulties in diagnosis, treatment-related side effects (primarily leukopenia), medication costs, and insurance barriers. Providers also identified facilitators of effective management such as subspecialty consultations, new therapeutic options, and pharmacist involvement in care coordination and medication management. Monitoring practices varied among providers, with most relying on serial PCR tests.

Providers identified several systemic challenges in managing CMV in kidney transplant recipients. These included ensuring appropriate follow-up, maintaining consistent laboratory monitoring, and obtaining insurance approval for newer therapies. Notably, while care coordination between specialties was identified as a challenge, providers emphasized the importance of interdisciplinary collaboration, particularly between transplant nephrology and infectious diseases, in optimizing CMV management. This suggests that while collaboration is recognized as crucial, implementing effective interdisciplinary care remains a challenge in practice. These systemic challenges may disproportionately affect minority populations, potentially explaining the observations in the quantitative analysis.

Our study’s strengths include its mixed-methods design and focus on minority populations, adding to the limited literature on CMV outcomes in these groups. However, the small sample size, particularly in the non-Hispanic white group, limits generalizability and reduces statistical power to detect significant difference. Future multicenter studies with larger, more balanced cohorts are needed to validate our findings. The retrospective nature may have introduced bias, including missing data and inconsistencies despite our efforts to minimize them. Qualitative data from providers, rather than from patients, may not fully capture patients’ perspectives on barriers to CMV management. The lack of standardized CMV management at our institution represents both a limitation and an opportunity to improve clinical practice. While reflecting real-world clinical decision-making, this variability may have influenced our outcomes.

Future research should focus on larger multicenter studies to validate these findings, explore disparities in CMV outcomes among minority kidney transplant recipients, and assess the impact of standardized protocols while addressing barriers to treatment access and adherence. Prospective studies with longer follow-up may elucidate the long-term effects of CMV on graft and patient survival across ethnic groups. Interventional studies targeting barriers, such as culturally tailored education and improved healthcare access, are also needed to develop strategies for reducing these disparities.

## Conclusion

Although limited by the small sample size, our study suggests potential disparities in CMV disease incidence, graft failure, and mortality among minority kidney transplant recipients, highlighting the unique challenges in managing CMV in this population. As transplant recipient demographics become increasingly diverse, addressing these disparities is crucial for promoting equitable care and optimizing long-term outcomes. Future efforts should prioritize standardized protocols, better interdisciplinary collaboration, and improved access to newer therapies to bridge the gap in CMV management and outcomes.
